# IRF5 is associated with adverse postoperative prognosis of patients with non-metastatic clear cell renal cell carcinoma

**DOI:** 10.18632/oncotarget.17777

**Published:** 2017-05-11

**Authors:** Qi Bai, Li Liu, Yu Xia, Jiajun Wang, Wei Xi, Yang Qu, Ying Xiong, Qilai Long, Jiejie Xu, Jianming Guo

**Affiliations:** ^1^ Department of Urology, Zhongshan Hospital, Fudan University, Shanghai 200032, China; ^2^ Department of Biochemistry and Molecular Biology, School of Basic Medical Sciences, Fudan University, Shanghai 200032, China

**Keywords:** clear cell renal cell carcinoma, IFN regulatory factor-5, nomogram, overall survival, recurrence free survival

## Abstract

**Background:**

IRF5 is one member of IRFs family, and is critical for host immunity and cell response. In the present study, we sought to search the clinical and prognostic value of IFR5 in patients with non-metastatic ccRCC.

**Results:**

IRF5 proved to be an adverse independent prognostic factor for overall survival (*p* < 0.001) and recurrence free survival (*p* = 0.002). The newly built nomograms could give better prediction for overall survival and recurrence free survival in ccRCC patients.

**Materials and Methods:**

We included 264 individuals who were diagnosed with non-metastatic clear cell renal cell carcinoma in the present study. Immunohistochemistry staining was performed on tissue microarrays to evaluate the IRF5 expression. *χ*^2^ test, Fisher's exact test, *t* test, Kaplan-Meier method and Cox proportional hazard model were applied to evaluate the prognostic value of IRF5. Two nomograms were constructed to predict clinical outcomes for ccRCC patients after surgery.

**Conclusions:**

IRF5 was an adverse independent prognostic factor for both overall survival and recurrence free survival in patients with non-metastatic ccRCC.

## INTRODUCTION

Renal cell carcinoma (RCC) represents 3% of all malignant tumors in adults [1]. The majority of RCC (70%) are classified as clear cell renal cell carcinoma (ccRCC) [2]. Despite most newly diagnosed RCC cases are early-stage and organ confined, approximately 25–30% of patients with RCC are presented with metastatic RCC (mRCC) at the time of diagnosis [3, 4]. Meantime 30% of the patients with localized disease will ultimately develop metastases even after the curative surgeries [5]. The nature history of mRCC is highly variable, with median overall survival of only 2 years [6–8]. We believe that continuous exploration of RCC biology and novel approaches to RCC management could help physicians in the process of surgical intervention and postsurgical medical intervention.

Clear cell renal cell carcinoma is a kind of heterogeneous disease. Multiple mediators, which were produced by the tumor itself or stromal compartments within the tumor microenvironment (TME) have profoundly influence on tumor behaviors. These mediators could serve as makers of tumor stage or novel targets for tumor treatment. Interferon regulatory factory 5 (IRF5) is a transcription factor that is responsible for type I IFN signaling [9] and multiple immune activities [10–12]. Increasing studies indicate that IRF5 could participate in the cellular response to stressors, including virus, DNA damage, and death receptor signaling [9, 13–15]. IRF5 could also regulate induction of multiple pro-inflammatory cytokines [16, 17], and then shaped the network of tumor immune microenvironment. Recently, the role of IRF5 in malignancy remains largely unknown and kind of controversial. Thus, better understanding of IRF5 may contribute an additional therapy target to the disease management and requires further attention.

The focus of the present study was to examine the clinical and prognostic importance of IRF5 in ccRCC. We had analyzed the expression of IRF5 by immunohistochemistry in ccRCC tissues and built two prediction models for overall survival (OS) and recurrence free survival (RFS), which integrated with other prognostic parameters, to refine individual risk stratification in non-metastatic ccRCC patients.

## RESULTS

### IRF5 expression and its association with pathological characteristics

**Figure 1:**
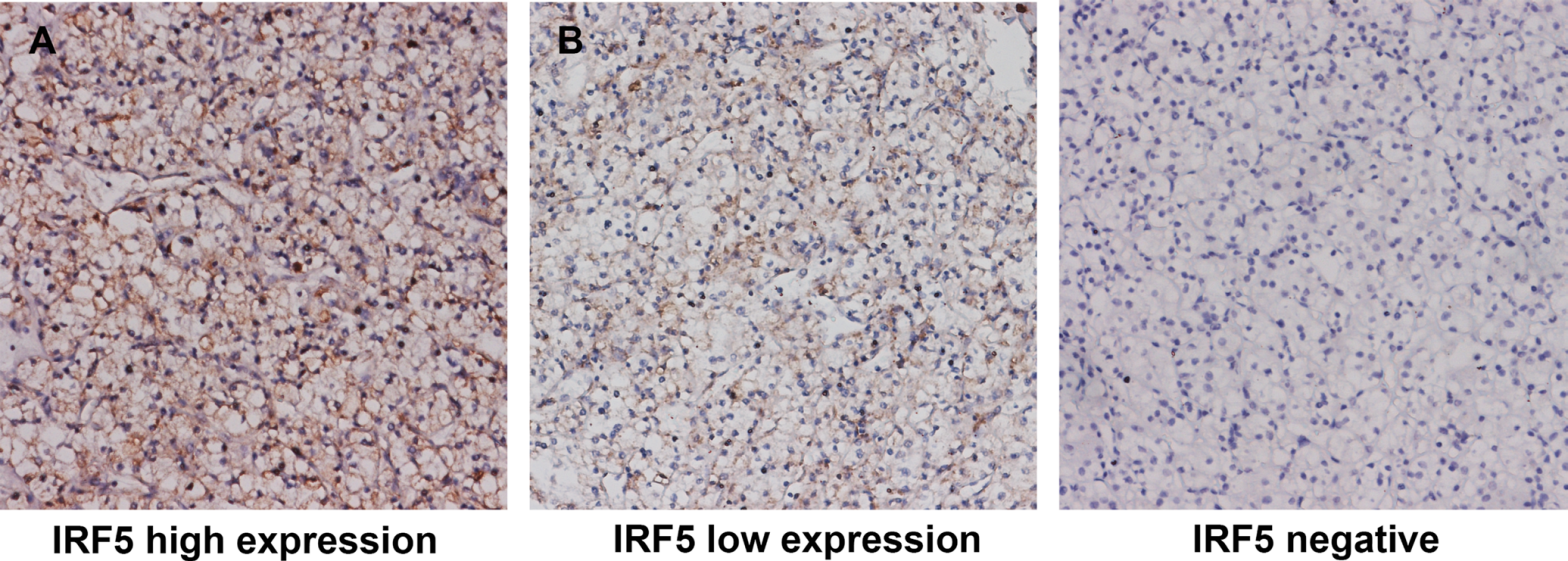
Representative pictures of IRF5 immunostaining High IRF5 expression in tumor tissue (**A**) low IRF5 expression in tumor tissue (**B**) negative IRF5 expression in tumor tissue. Original magnification: ×200.

IRF5 positive staining in ccRCC predominantly located in the nuclear and cytoplasm (Figure 1). According to IRF5 expression, 30.3% (80/264) of patients were defined as high IRF5 expression. Table 1 showes detail characteristic of the study population and its correlation with IRF5. The median follow-up time was 99.7 (rang: 3–120) months. IRF5 expression was not associated with age, gender ECOG PS, T stage, Fuhrman grade or necrosis.

**Table 1:** Correlation between IRF5 expression and clinical characteristics in localized ccRCC patients

Variables	All patients		IRF5 expression		
	No.	%	Low	High	p*
Age at surgery. yr					0.874
Mean ± SD	55.1 ± 12.9		55.2 ± 13.2	54.9 ± 12.4	
Gender					0.895
Female	81	30.7	56	25	
Male	183	69.3	128	55	
ECOG PS					0.470
0	190	72.0	130	60	
≥ 1	74	28.0	54	20	
Surgery					0.362
Partial nephrectomy	19	7.2	15	4	
Radical nephrectomy	245	92.8	169	76	
Pathological T stage					0.730
pT1	185	70.0	130	55	
pT2	19	7.2	12	7	
pT3	58	22.0	40	18	
pT4	2	0.8	2	0	
Fuhrman nuclear grade					0.830
1	29	11.0	19	10	
2	204	77.3	144	60	
3	31	11.7	21	10	
Necrosis					0.686
Absent	231	87.5	162	69	
Present	33	12.5	22	11	

Abbreviations: ccRCC: clear cell renal cell carcinoma. ECOG PS: Eastern Cooperative Oncology Group performance status. SD: standard deviation.

*t test, χ^2^ test or Fisher's exact test were performed. *p* < 0.05 was regard as statistically significant.

### Kaplan-Meier survival analysis and subgroup analysis

**Figure 2:**
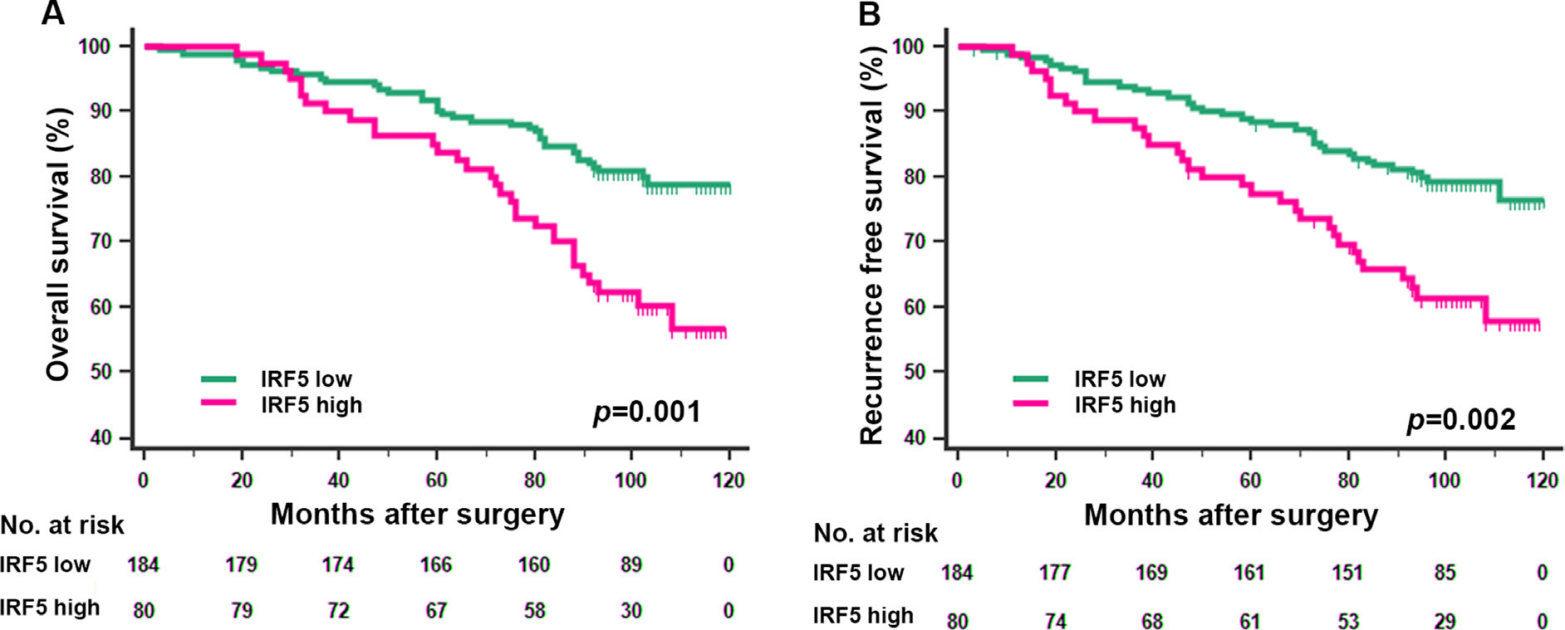
Kaplan-Meier analyses for overall survival and recurrence free survival of patients with ccRCC according to IRF5 expression Overall survival according to IRF5expression in non-metastatic ccRCC (**A**) recurrence free survival according to IRF5expression in non-metastatic ccRCC (**B**) *p*-value was calculated by Log rank test, *p* < 0.05 was regarded as statistically significant.

**Figure 3:**
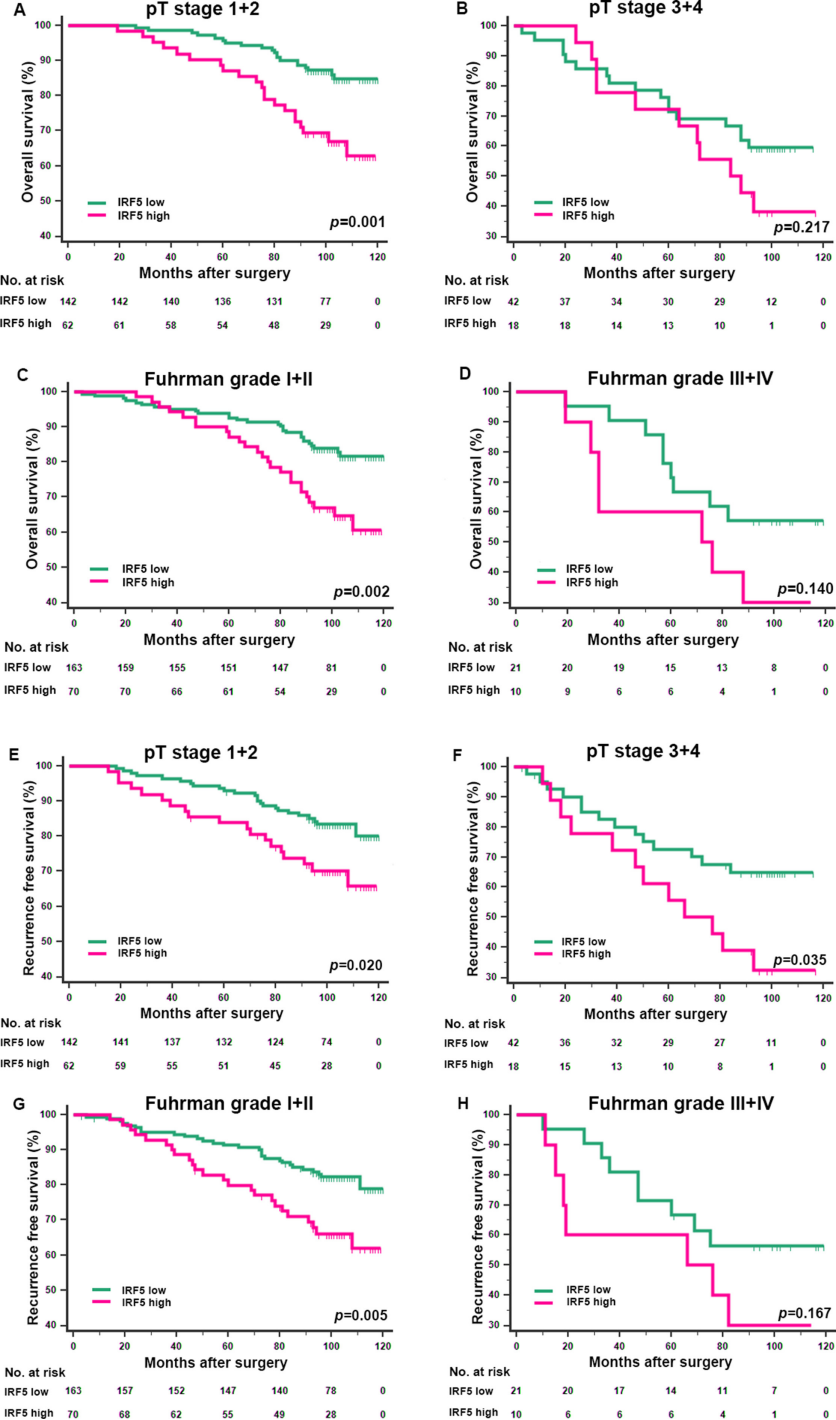
Kaplan-Meier analyses for overall survival and recurrence free survival of patients in pT stage and Fuhrman grade subgroups Overall survival for patients in the pT stage (1+2) group (**A**) pT (3+4) stage group (**B**) Fuhrman grade (I+II) group (**C**) and Fuhrman grade (III+IV) group (**D**) according to IRF5 expression; recurrence free survival for patients in the pT stage (1+2) group (**E**) pT stage (3+4) group (**F**) Fuhrman grade (I+II) group (**G**) and Fuhrman grade (III+IV) group (**H**) according to IRF5 expression; *p*-value was calculated by Log rank test, *p* < 0.05 was regarded as statistically significant.

The Kaplan-Meier survival method was applied to analyze OS and RFS according to IRF5 expression. Patients with high IRF5 expression had significantly poorer OS (*p* = 0.001) and RFS (*p* = 0.002) (Figure 2A, 2B) than those with low IRF5 expression. In the further study, we analyzed IRF5 expression in different T stage and Fuhrman grade subgroups. We found that the unfavorable prognostic value of IRF5 in lower risk ccRCC patients. For the OS, the low/high expression of IFR5 could only distinguish patients with dismal outcome in T stage (1+2) group (Figure 3A, *p* = 0.001) and Fuhrman grade (I+II) group (Figure 3C, *p* = 0.002) but not in T stage (3+4) group (Figure 3B, *p* = 0.217) or Fuhrman grade (III+IV) group (Figure 3D, *p* = 0.140). And for RFS, the prognostic value of IRF5 is significant in patients in both T stage (1+2 and 3+4) groups (Figure 3E, 3F, *p* = 0.020 and *p* = 0.035 respectively) and Fuhrman grade (I+II) group (Figure 3G, *p* = 0.005) but not in Fuhrman grade (III+IV) group (Figure 3H, *p* = 0.167).

### Cox regression analyses

To investigate if IRF5 was an independent prognostic factor for OS and RFS, univariate and multivariate Cox regression models were used. We analyzed T stage, Fuhrman grade, necrosis and ECOG PS in univariate analysis. The variables have long been used as predictors for patients with ccRCC. After univariate analysis, we included the statistically significant parameters into multivariate analysis (Table 2). The hazard ratio of IRF5 expression was 2.21 (95% CI: 1.38–3.55, *p* < 0.001) and 2.10 (95% CI: 1.30–3.37, *p* = 0.002) for OS and RFS respectively. The results showed that IRF5 could serve as an adverse independent prognostic factor for both OS and RFS.

**Table 2:** Univariate and multivariate cox regression analyses for overall survival and recurrence free survival in localized ccRCC patients

Variables	Univariate analysis		Multivariate analysis	
	HR(95% CI)	p*	HR(95% CI)	p*
Overall survival				
pT stage		**< 0.001**		**< 0.001**
pT2 *vs* pT1	3.34 (1.59–7.00)	**0.001**	3.35 (1.58–7.12)	**0.002**
pT3 *vs* pT1	3.43 (2.04–5.77)	**< 0.001**	3.35 (1.95–5.76)	**< 0.001**
pT4 *vs* pT1	141.81 (26.42–761.37)	**< 0.001**	292.79 (51.87–1652.60)	**< 0.001**
Fuhrman grade		**< 0.001**		**0.003**
2 *vs* 1	1.86 (0.67–5.16)	0.419	1.34 (0.48–3.79)	0.578
3 *vs* 1	5.35 (1.79–16.01)	**0.002**	3.86 (1.24–11.98)	**0.019**
Necrosis (present *vs* absent)	2.82 (1.61–4.89)	**< 0.001**	1.75 (0.94–3.25)	0.075
ECOG PS (≥ 1 *vs* 0)	1.165 (0.70–1.95)	0.300	–	–
IRF5 (high *vs* low)	2.21 (1.38–3.55)	**< 0.001**	2.56 (1.51–3.99)	**< 0.001**
Recurrence-free survival				
pT stage		**< 0.001**		**< 0.001**
pT2 *vs* pT1	3.34 (1.60–6.99)	**0.001**	3.33 (1.57–7.04)	**0.002**
pT3 *vs* pT1	3.12 (1.86–5.22)	**< 0.001**	3.19 (1.86–5.48)	**< 0.001**
pT4 *vs* pT1	42.18 (5.22–340.41)	**< 0.001**	85.04 (10.14–712.85)	**< 0.001**
Fuhrman grade		**< 0.001**		**0.006**
2 *vs* 1	1.88 (0.68–5.21)	0.224	1.44 (0.51–4.05)	0.492
3 *vs* 1	5.34 (1.78–15.99)	**0.003**	3.78 (1.22–11.67)	**0.021**
Necrosis (present *vs* absent)	3.12 (1.82–5.35)	**< 0.001**	2.14 (1.18–3.89)	**0.012**
ECOG PS (≥ 1 *vs* 0)	1.04 (0.62–1.76)	0.891	–	–
IRF5 (high *vs* low)	2.10 (1.30–3.37)	**0.002**	2.29 (1.42–3.71)	**0.001**

Abbreviations: ECOG PS: Eastern Cooperative Oncology Group performance status; HR: hazard ratio; CI: confidence interval; ccRCC: clear cell renal cell carcinoma.

*Data obtained from the Cox proportional hazards model; *p* < 0.05 was regard as statistically significant.

### Nomogram for predicting OS and RFS in ccRCC

**Figure 4:**
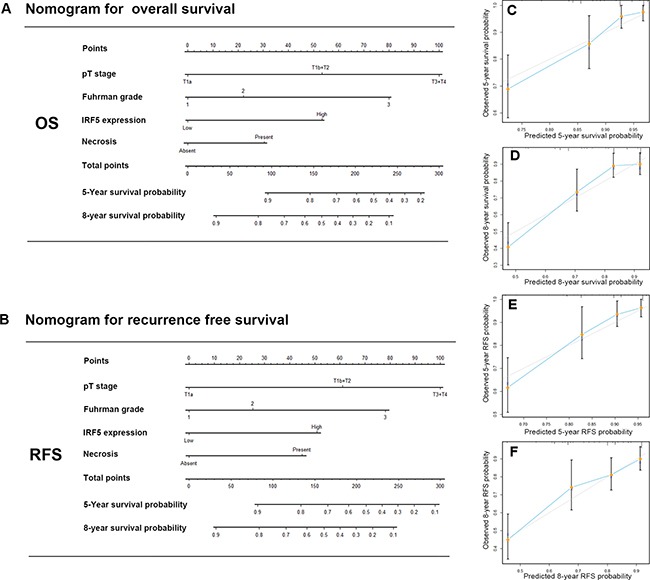
Nomogram for predicting 5- and 8-year overall survival and recurrence free survival in patients with ccRCC Nomogram for predicting 5- and 8- year OS (**A**) Nomogram for predicting 5- and 8- year RFS (**B**) Calibration plot for nomogram predicted and observed 5-year overall survival rate (**C**) and 8-year overall survival rate (**D**) Calibration plot for nomogram predicted and observed 5-year recurrence free survival rate (**E**) and 8-year recurrence free survival rate (**F**) higher total point indicated a more adverse outcome probability;. Line of dashes: ideal model, vertical bars: 95% confident interval.

On the basis of results above, we attempted to build a prediction model that incorporating of IRF5 expression and other prognostic parameters to give a better stratification of clinical outcomes. The prognostic parameters include T stage, Fuhrman grade and presence of necrosis. We constructed two nomograms to predict OS and RFS at 5 and 8 years after surgery (Figure 4A, 4B). Higher total point indicated worse outcome. The calibration plots of the nomograms were shown for OS (Figure 4C, 4D) and RFS (Figure 4E, 4F). The Harrell's c-indices for OS and RFS were 0.78 (95% CI, 0.72–0.83) and 0.77 (95% CI, 0.71–0.83) respectively, higher than the combination of other prediction factors except IRF5, 0.72 (95% CI, 0.66–0.78) for OS and 0.70 (95% CI: 0.64–0.76) for RFS. So we believed that the newly constructed model could give a better prediction for OS and RFS.

## DISCUSSION

Currently, several clinical and/or genomic factors have been proposed to identify RCC patients who are at greater risk of disease progression [18–22]. In the present study, we examined the prognostic value of IRF5 in non-metastatic ccRCC patients. The results presented here provide for the first time that high IRF5 expression correlated with ccRCC development and progression. We also found that IRF5 could further stratify the patients in lower risk group like pT stage (1 and 2) or Fuhrman grade (1 and 2). Numerous biomarkers have been investigated, but little of them could improve the predictive accuracy of the current prognostic systems. The present nomograms which integrated IRF5 with other prognostic parameters could give a better risk stratification for OS and RFS in non-metastatic ccRCC patients.

IRFs have multiple functions and abnormal expression could lead to aberrant biological cell behaviors [23]. Currently, little is known about their roles in ccRCC development and progression. IRF5 is one member of IRFs family, and is critical for host immunity and the cellular response to extracellular stressors [10, 12]. IRF5 also plays essential roles in the cell growth, cell cycle, innate antiviral and inflammatory responses [9, 13, 17, 24]. Increasing studies support the notion that inflammation and cancer immune responsiveness may share a common determinism [25]. IRF5 proved to be associated with some autoimmune diseases like systemic lupus erythematosus [26, 27] and rheumatoid arthritis [28].

In fact, the role of IRF5 in tumor genesis remains controversial. As IRF5 can induce *p21, Bak, Bax, and Caspase 8*, making it a potential candidate of tumor-suppressor [29–31]. In immortalized tumor cell lines and primary samples from patients with hematological malignancies, IRF5 expression is always absent or significantly decreased [13]. It is reported that in hepatocellular carcinoma and gastric cancer, IRF5 expression is down-regulated due to gene promoter hyper-methylation [32, 33]. However, Michele et al. showed that in thyroid cancers cells, IRF5 displays tumor-promoting property [34]. And in the present results, we also defined IRF5 as an independent risk factor for ccRCC progression.

Accumulating evidence has unveiled robust and supportive contributions of tumor microenvironment (TME) to the survival, self-renewal and tumorigenic activities of tumors [35, 36]. However how the functional and phenotypic heterogeneity of tumor itself, in turn, impacts the pathophysiological activities of TEM remains unknown. IRF5 mediates induction of multiple pro-inflammatory cytokines, such as IL-1, IL-6, and TNF-a [16] and these cytokines had proved to be indication of a worse outcome in quite a few tumors [37–39]. In addition, Masahisa et al. found that resistance to cytotoxic chemotherapy renders cancer stem cells (CSCs) ability to create immunosuppressive microenvironments through IRF5 pathways [40]. Tumor associated macrophages (TAMs) are one of the major populations of tumor infiltrating immune cells and have been shown to play critical roles in promoting the tumorigenic activities [41–43]. And in our previous study, we found polarized TAMs were novel independent prognosticator in patients with ccRCC [44]. Constitutive activation of IRF5 has been identified as indispensable for triggering M-CSF production and the resultant monocyte infiltration and differentiation of M2-type macrophages in tumor tissues [40]. RCC monocytes express a mixture of both M1 and M2 gene, and pro-inflammatory monocytes in RCC display a tumor-promoting phenotype [45], which means the inflammatory microenvironment may be a hallmark of ccRCC. Recently, our knowledge of IRF5 in ccRCC remains limited and the mechanism responsible for carcinogenesis is still lacking and merits further research.

In conclusion, we have revealed that IRF5 expression could serve as an adverse independent prognostic factor in non-metastatic ccRCC. Higher IRF5 expression indicated worse clinical outcomes than the counterparts. Moreover, the prediction model we built in the present study could further stratify the patients with different outcomes. Above all, we have reason to believe that IRF5 might promote ccRCC progression. Limitations of the present study are the retrospective design and only non-metastatic disease are involved. A multicenter and prospective study is needed to validate the results.

## MATERIALS AND METHODS

### Patient selection

Medical records of patients who were treated in Zhongshan Hospital, Fudan University (Shanghai, China) between Jan 2005 and Jun 2007 were reviewed. We retrospectively recruited 264 patients who underwent radical nephrectomy or nephron-sparing surgery at Zhongshan Hopital. Following clinicopathological characters include age, sex, tumor size, TNM stage, pathological data and ECOG PS were collected from the database of the institution. The corresponding department approved the access to medical records. Tumor stage and postoperative histopathological type were determined according to the 2010 AJCC TNM classification [46]. The inclusion criteria were as follows: (1) the histopathological type should be clear cell RCC, (2) no history of neoadjuvant therapy before surgery, (3) no history of other malignancy before, (4) patients with lymph node or distant metastasis were excluded from the present study. We invited a practiced pathologist to re-evaluated all sections from nephrectomy samples to verify the Fuhrman grades, histology type, and presence of necrosis. We also excluded the patient whose corresponding tissue was mostly necrosis (> 80%) or histopathology features represented a combination of clear cell RCC and other RCC type. Patients were followed up every 6 months or earlier for the first 2 years right after the nephrectomy and every 12 months thereafter. The study was approved Clinical Research Ethics Committee of Zhongshan Hospital, Fudan University with the approval number B2015-030 in Feb 2015. Written, informed consent was obtained from each individual enrolled in the study.

### Immunohistochemistry and evaluation

We performed immunohistochemistry staining on tissue microarrays (TMAs). The TMAs construction was described previously[47]. We used anti-IRF5 antibody (ab181553, Abcam, Cambridge, MA, USA) as the primary antibody in the procedure. Two independent pathologists who were blind to the clinical outcome, were asked to evaluate the staining intensity and extent. The intensity score was graded as 0 (negative), 1 (weak), 2 (moderate), and 3 (strong); the extent score was calculated by the percentage of the positive cells (0%–100%). We multiplied the staining intensity and extent to calculate the expression score ranging from 0 to 300. X-Tile software (Yale University School of Medicine, New Haven, CT, USA) was used to determine the cutoff point of high/low expression through minimum *p* value method based on patients’ OS information, and 180 was selected as the cutoff point.

### Statistical analyses

We assessed correlations between IRF5 expression and patient clinical characteristics with *t* test, χ^2^ test or Fisher's exact test. Kaplan-Meier survival curves were established and statistical significance was analyzed by log-rank test. Cox proportional hazard models were used to analyze the effect of patient characteristics, clinical features and IRF5 expression on OS and RFS. All the statistical tests were two sided and *p* < 0.05 was considered statistical significant. We generated two nomogram models using R software with “rms” package (R Foundation for Statistical Computing, Vienna, Austria). Parameters with *p <* 0.1 level in multivariate analyses were included in nomogram. Calibration plots for OS and RFS were constructed to exhibit the performance of the present model. Harrell's concordance indices (c-indices) were calculated to test the prognostic accuracy. All statistical analyses above were performed using SPSS version 19.0 (SPSS Inc., IL, Chicago, USA) and R software with “rms” package (R Foundation for Statistical Computing, Vienna, Austria).
